# Epidemiologic changes of a longitudinal surveillance study spanning 51 years of scrub typhus in mainland China

**DOI:** 10.1038/s41598-024-53800-y

**Published:** 2024-02-07

**Authors:** Pei-Ying Peng, Hui-Ying Duan, Lei Xu, Lin-Tao Zhang, Ji-Qin Sun, Ya Zu, Li-Juan Ma, Yan Sun, Ting-Liang Yan, Xian-Guo Guo

**Affiliations:** 1 Institute of Microbiology of Qujing Medical College, Qujing, 655011 Yunnan Province China; 2 Department of Clinical Laboratory, Qujing Second People’s Hospital, Qujing, 655011 Yunnan Province China; 3 Institute of Pathogens and Vectors, Yunnan Provincial Key Laboratory for Zoonosis Control and Prevention, Dali University, Dali, 671000 Yunnan China

**Keywords:** Infectious diseases, Public health, Epidemiology

## Abstract

Scrub typhus may be one of the world’s most prevalent, neglected and serious, but easily treatable, febrile diseases. It has become a significant potential threat to public health in China. In this study we used national disease surveillance data to analyze the incidence and spatial–temporal distribution of scrub typhus in mainland China during 1952–1989 and 2006–2018. Descriptive epidemiological methods and spatial–temporal epidemiological methods were used to investigate the epidemiological trends and identify high-risk regions of scrub typhus infection. Over the 51-year period, a total of 182,991 cases and 186 deaths were notified. The average annual incidence was 0.13 cases/100,000 population during 1952–1989. The incidence increased sharply from 0.09/100,000 population in 2006 to 1.93/100,000 population in 2018 and then exponentially increased after 2006. The incidence was significantly higher in females than males (χ^2^ = 426.32, *P* < 0.001). Farmers had a higher incidence of scrub typhus than non-farmers (χ^2^ = 684.58, *P* < 0.001). The majority of cases each year were reported between July and November with peak incidence occurring during October each year. The trend surface analysis showed that the incidence of scrub typhus increased gradually from north to south, and from east and west to the central area. The spatial autocorrelation analysis showed that a spatial positive correlation existed in the prevalence of scrub typhus on a national scale, which had the characteristic of aggregated distribution (*I* = 0.533,* P* < 0.05). LISA analysis showed hotspots (High–High) were primarily located in the southern and southwestern provinces of China with the geographical area expanding annually. These findings provide scientific evidence for the surveillance and control of scrub typhus which may contribute to targeted strategies and measures for the government.

## Introduction

Scrub typhus was first described by Hashimoto from Japan in 1899 and it is also known as tsutsugamushi disease. The name tsutsugamushi is derived from two Japanese words: tsutsuga, meaning something small and dangerous, and mushi, meaning creature. The infection is called scrub typhus because it generally occurs after exposure to areas with secondary (scrub) vegetation^1^. However, it has recently been found that the disease can also be prevalent in such areas as sandy beaches, mountain deserts, and equatorial rain forests^2,3^. It is a zoonotic infectious disease caused by *Orientia* spp. and humans can be exposed to this bacterium through bites of infected larval-stage trombiculid mites^4^. The clinical manifestations of scrub typhus can range from unapparent or atypical febrile illness to life-threatening symptoms including acute hearing loss and multiple organ failure^5,6^. As an emerging and re-emerging disease with unspecific clinical manifestations, scrub typhus has been neglected and often misdiagnosed. Moreover, there are no long-lasting, broadly-protective vaccines available against scrub typhus at present^7^, and early diagnosis and treatment can significantly reduce the complication and fatality rate.

Scrub typhus is widely prevalent in the ‘tsutsugamushi triangle’, north to northern Japan and eastern Russia, south to northern Australia and west to Pakistan and Afghanistan^8^. The tsutsugamushi triangle is home to more than half the world’s population, with 2 billion people at risk and 1 million cases of scrub typhus occurring per year^9,10^. Scrub typhus remains a serious public health problem in China. Recent evidence from China showed that scrub typhus has expanded to all the provinces in mainland China and the incidence of scrub typhus has increased rapidly in an unprecedented manner, which could signal the re-emergence of this neglected tropical disease^10,11^. Previous studies in China have investigated the epidemiological features of scrub typhus in some provinces or cities aimed at areas of high incidence^9,12,13^, but the nationwide trends in reported cases have yet to be adequately characterized. Moreover, some studies on the epidemiological characteristics of scrub typhus at the national level are also short-term studies^11,14,15^.

This paper comprehensively and systematically describes the long-term (51-year) epidemiologic characteristics and spatiotemporal analysis of scrub typhus, and identifies some high-risk areas in the country, using data spanning from 1952 to 2018 (1952–1989 and 2006–2018). This information would be helpful for health administration officers and public health workers with regard to the implementation of effective intervention measures targeted toward high-risk areas and populations.

## Results

### Descriptive epidemiology

**Figure 1:**
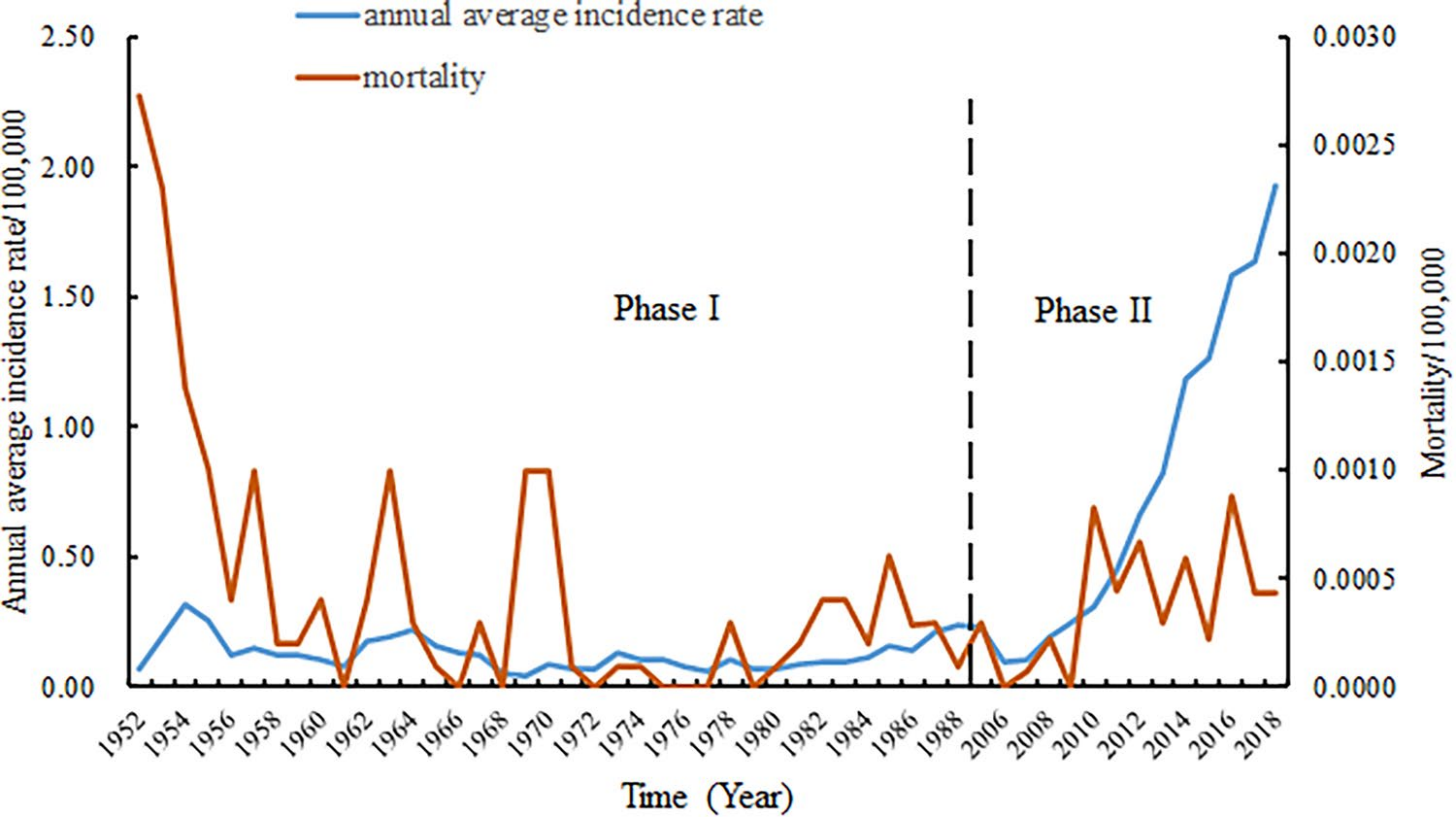
Annual average incidence rate and mortality of scrub typhus in China from 1952 to 1989 (phase I) and 2006–2018 (phase II).

**Figure 2:**
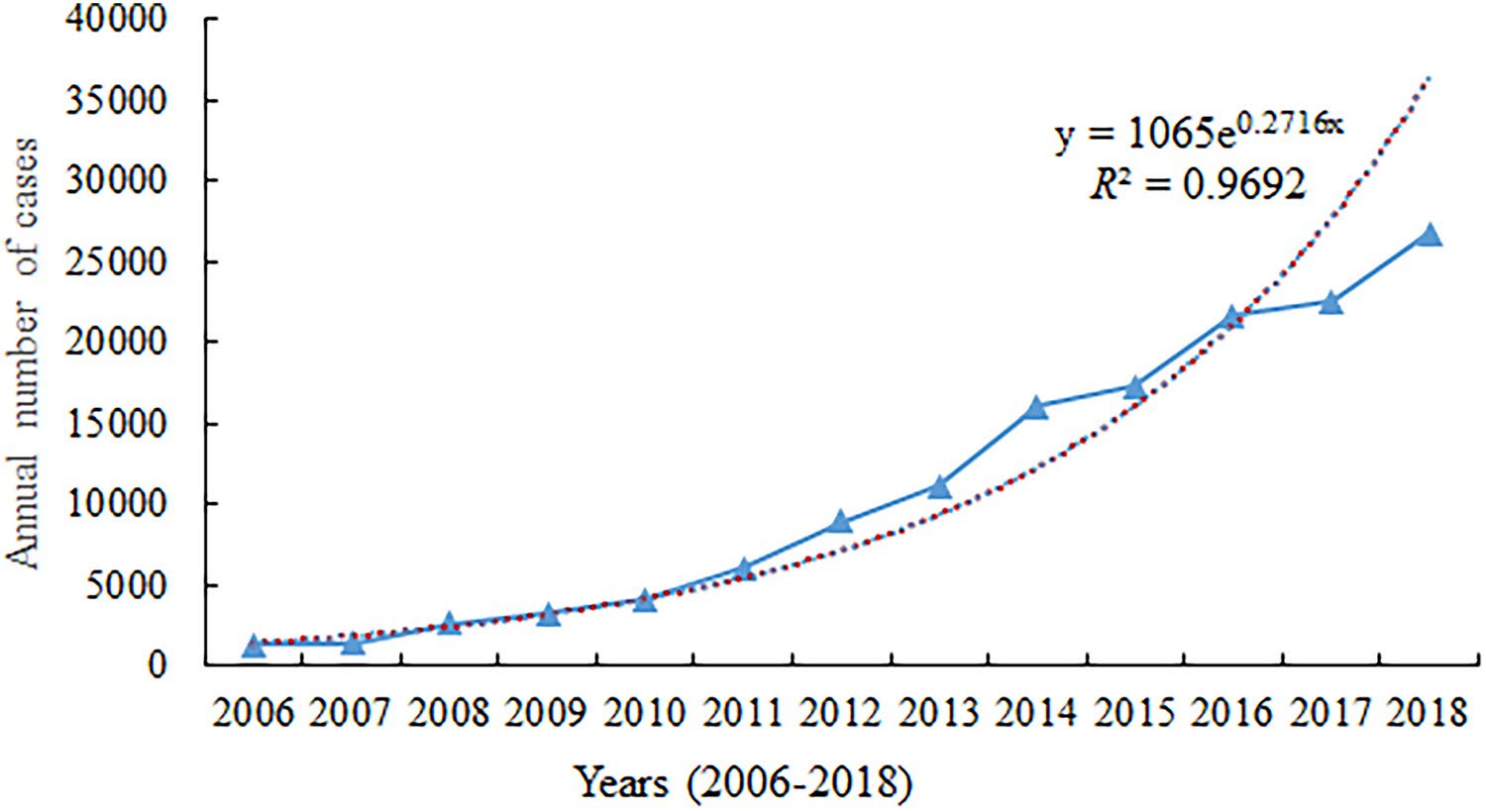
The total number of annual cases (blue line with the triangle) fit with an exponential growth function (red broken line).

**Figure 3:**
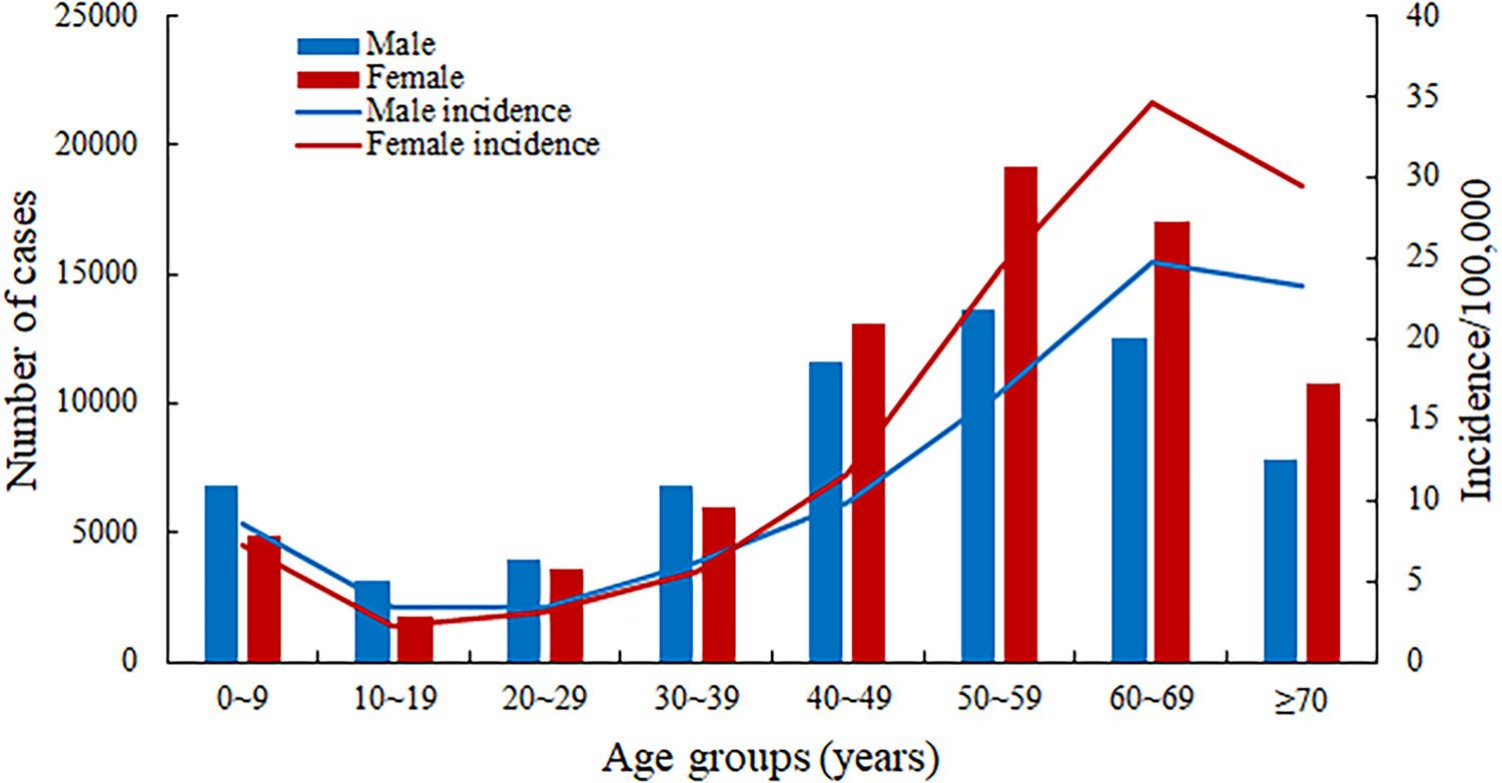
Age and sex distribution of reported scrub typhus cases in China, 2006–2018.

A total of 182,991 cases of scrub typhus were reported in mainland China in 1952–1989 and 2006–2018 during the 51 years covered in this study. During 1952–1989, the occurrence of scrub typhus remained at a stable, low level, with an average annual incidence rate of 0.13 (95% CI 0.11–0.15) per 100,000 population. Since 2006, the annual morbidity has sharply increased 21 times from 0.09/10 0000 to 1.93/10 0000 with a 35% annual growth rate (95% CI 30–38%) (Table 1, Fig. 1). The total number of annual cases increased exponentially (*R*^2^ = 0.97) (Kolmogorov–Smirnov test, *P* = 0.924 > 0.05) (Fig. 2). Of these, 46.54% were male and 53.46% were female. The female incidence rates were higher than male incidence rates in persons 40–49, 50–59, 60–69, and ≥ 70 years. The highest incidence was among those 60–69 years (Fig. 3). Regarding occupation, 76.22% of scrub typhus cases were farmers, followed by preschoolers (6.38%), unemployed and the retired (6.36%), workers (4.23%) and students (4.04%) (Table 1). Case-patients > 40 years of age were the most common (74.06%); the highest proportion was in the age group aged 50–59 years old (22.95%), 60–69 years old (20.73%), and 40–49 years old (17.33%). The 60–69 and ≥ 70-year-old age groups had a relatively high incidence (29.67 cases/100,000 and 26.55 cases/ 100,000, respectively) (Table 1). The main endemic areas of scrub typhus were located in rural areas, which accounted for 88.0% of the reported cases (Table 1).

**Table 1:** Demographic characteristics of reported scrub typhus cases in mainland China, 2006–2018.

Parameters	Cases	Incidence per 100,000	Proportion	Male% vs. Female%	*P* value
Total	142,849	10.66	100.00	50:50	
Year					
2006	1244	0.09	0.87	51:49	
2007	1332	0.10	0.93	53:47	
2008	2592	0.19	1.81	53:47	
2009	3235	0.24	2.26	51:49	
2010	4085	0.30	2.86	50:50	
2011	6020	0.45	4.21	48:52	
2012	8921	0.66	6.25	47:53	
2013	11,104	0.82	7.77	44:56	
2014	16,032	1.19	11.22	45:55	
2015	17,280	1.28	12.10	46:54	
2016	21,691	1.60	15.18	46:54	
2017	22,556	1.62	15.79	46:54	
2018	26,757	1.93	18.73	46:54	
Age					
0–9	11,702	7.99	8.19	58:42	
10–19	4962	2.84	3.47	64:36	
20–29	7593	3.32	5.32	52:48	
30–39	12,811	5.95	8.97	53:47	
40–49	24,753	10.75	17.33	47:53	
50–59	32,784	20.48	22.95	42:58	
60–69	29,609	29.67	20.73	42:58	
≥ 70	18,635	26.55	13.05	42:58	
Gender					< 0.05^#^
Male	66,481	9.68	46.54	–	
Female	76,368	11.70	53.46	–	
Sex ratio	0.87		–	–	
Occupation					
Farmers	108,880	–	76.22	42:58	
Workers	6042	–	4.23	59:41	
Students	5774	–	4.04	65:35	
Preschoolers	9116	–	6.38	63:37	
The unemployed and the retired	9082	–	6.36	42:58	
Others	3955	–	2.77	56:44	
Geographic area*					< 0.001^#^
Rural	123,789	18.36	88.00	43:57	
Urban	17,090	2.57	12.00	52:48	

^#^*P* value calculated by use of *χ*^2^ test.

*Two columns (Rural and Urban) do not add up to equal the total because of missing data

### Time-series analysis

**Figure 4:**
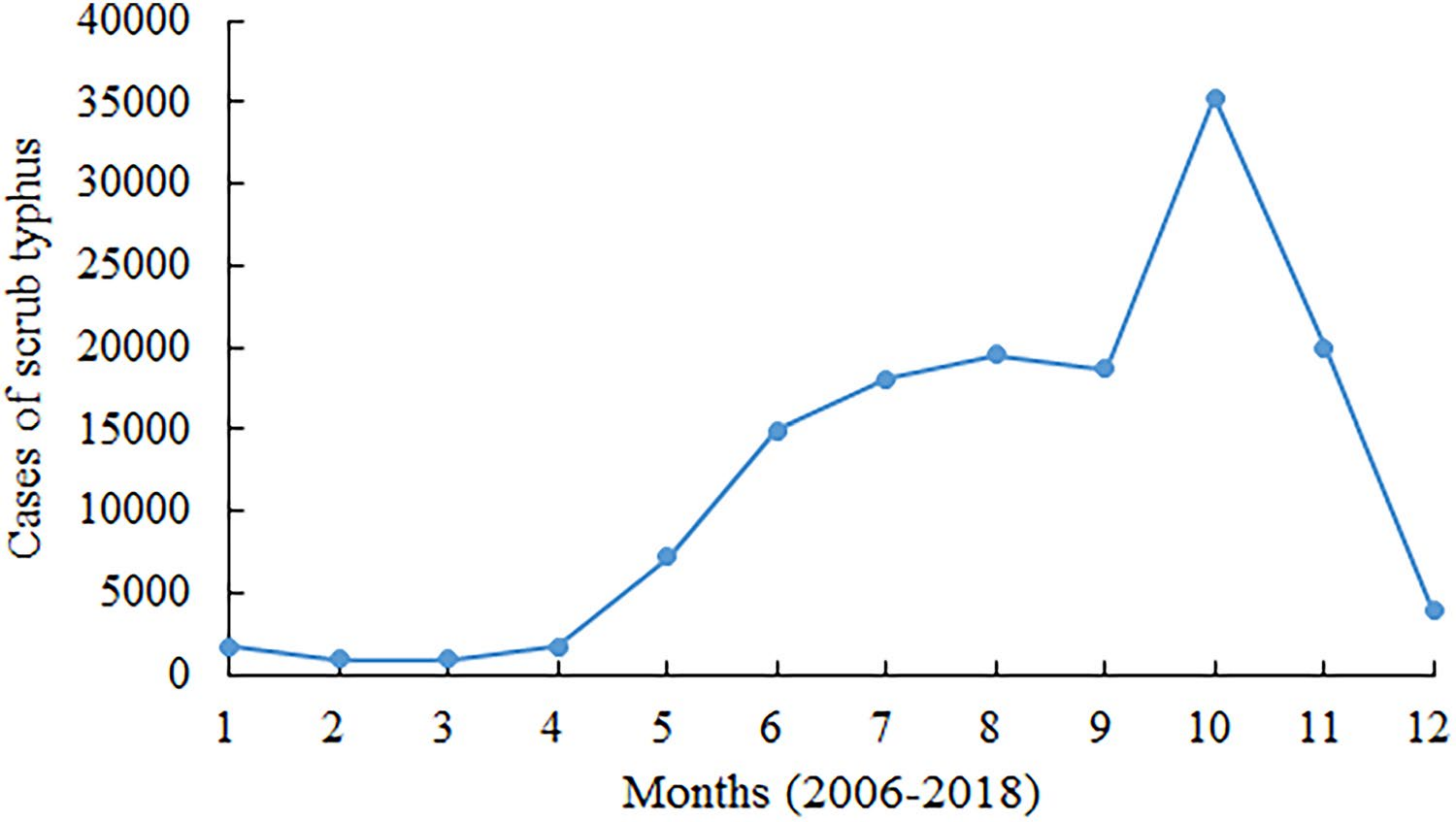
Seasonal patterns in reported scrub typhus cases in mainland China, 2006–2018.

Overall, 40,142 cases and 117 deaths were reported during period one (from 1952 to 1989), with an annualized average incidence of 0.13 cases/100,000 population and a fatality rate of 0.29% (Fig. 1). There were 142,849 cases and 69 deaths in 2006–2018. The yearly cases and the yearly incidence increased year by year. The yearly cases increased from 1244 to 26,757 in 2006–2018. The yearly incidence increased from 0.09 per 100,000 population to 1.93 per 100,000 population in 2006–2018 (Table 1). The seasonal pattern of scrub typhus cases begins to increase dramatically in April through September, before reaching a peak in October and returning to low, constant levels of transmission in the winter months of December through March (Fig. 4).

**Figure 5:**
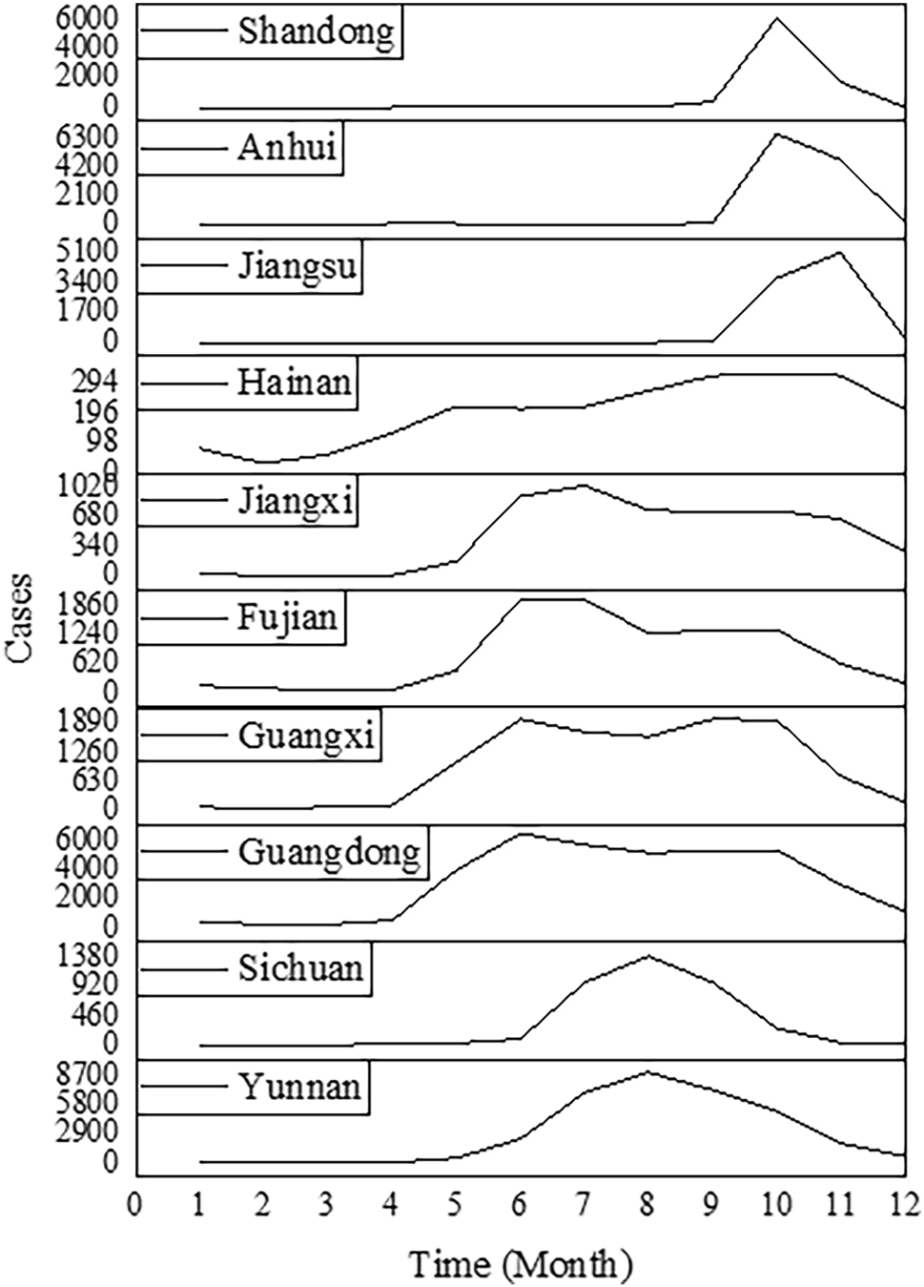
Seasonal distributions of scrub typhus cases in different regions, China, 2006–2018.

Differences in the seasonal trends of incidence between northern and southern provinces were identified. In Fujian, Guangdong, and Guangxi, scrub typhus occurred mainly in June to July and September to October, demonstrating a bimodal appearance of two peaks. In Yunnan, the number of cases demonstrated a single large peak in July or August. In contrast, scrub typhus cases demonstrated a single large peak in October or November in the northern provinces (Shandong, Anhui, and Jiangsu) each year (Fig. 5).

### Spatial distribution analyses

All of the 31 provinces (municipalities) in mainland China reported scrub typhus during 2006–2018. The top five provinces regarding the total number of scrub typhus cases were Guangdong, Yunnan, Anhui, Guangxi and Fujian, and the top five provinces regarding the annual average incidence rate per capita were Yunnan, Guangdong, Hainan, Fujian and Guangxi (Table 2). The provinces with less than 10 cases were Shanxi (9), Inner Mongolia (8), Jilin (6), Tibet (5), Xinjiang (4), Shanghai (2), Qinghai (1) and Ningxia (1).

**Table 2:** Reported cases and incidence of scrub typhus in the top 10 provinces, China, 2006–2018.

Province	Total number of cases	Annual average incidence rate/100,000
Guangdong	38,439	2.83
Yunnan	35,420	5.93
Anhui	12,198	1.58
Guangxi	11,718	1.96
Fujian	9935	2.07
Jiangsu	9299	0.91
Shandong	8642	0.69
Jiangxi	5509	0.95
Sichuan	4053	0.39
Hainan	2436	2.16

### Trend surface analysis

**Figure 6:**
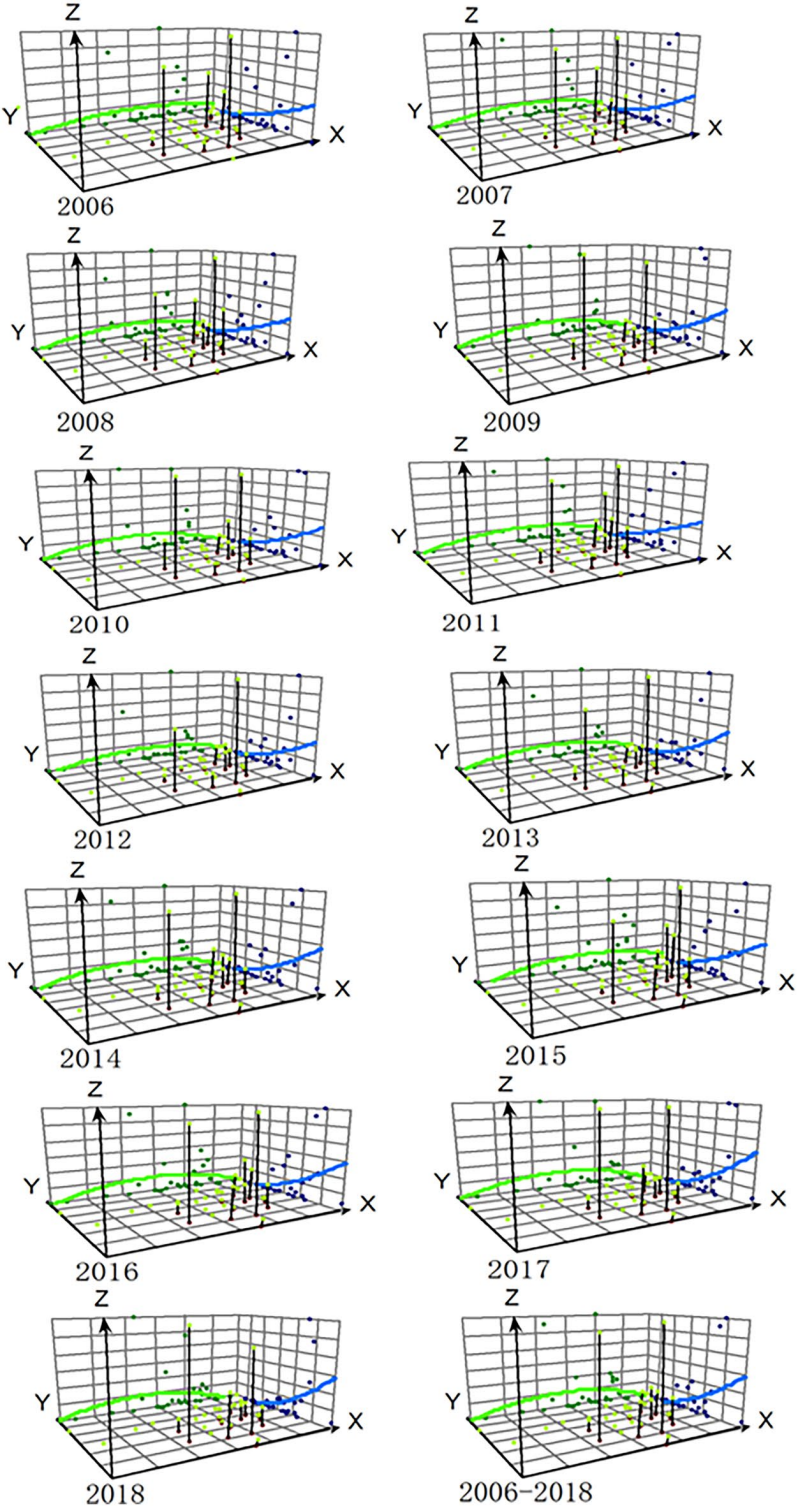
Three-dimensional trend surface analysis on incidences of scrub typhus in China, 2006–2018. This map was created by ArcGIS software (version 10.6, ESRI Inc.; Redlands, CA, USA). Homepage of ArcGIS software was https://www.esri.com/. *Note* The X-axis represents longitude; The Y-axis is latitude; The Z-axis shows incidence; the green line represents the incidence trend of the west–east direction, and the blue line represents the incidence trend of the north–south direction.

The trend surface analysis was employed to identify the geographic trends of the incidence (Fig. 6). A coordinate system was created (one axis for each direction with X for West–East and Y for South–North). The projections of incidence rates (Z axis) reflected the variation trend of West–East and South–North. The trend surface analysis showed that the incidence of scrub typhus increased gradually from north to south, and from east and west to the central area.

### Spatial autocorrelation of scrub typhus in China

**Figure 7:**
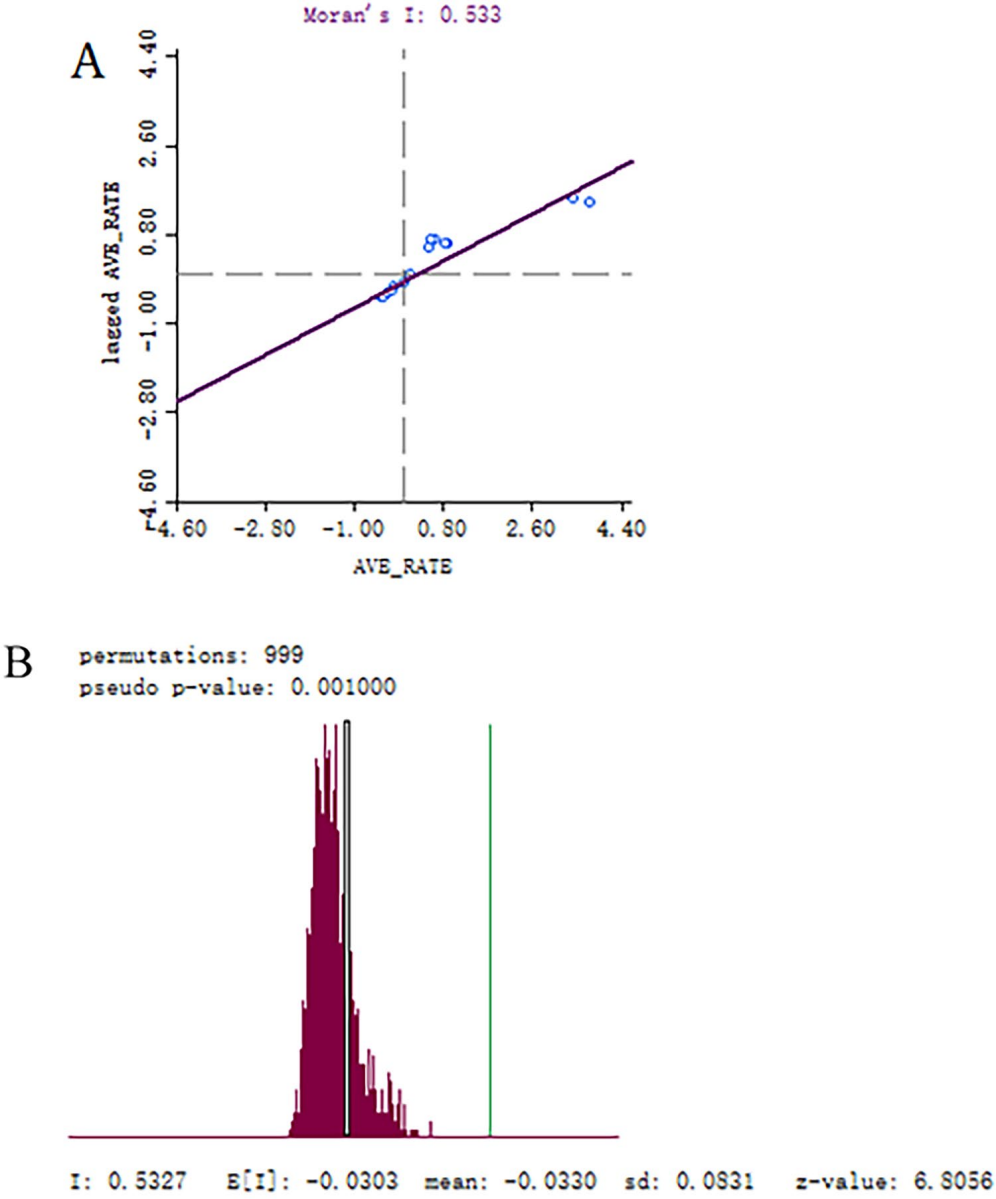
Global spatial autocorrelation analysis for annual average incidence of scrub typhus in China from 2006 to 2018. (**A**) Moran’s scatter plot for annualized average incidence of scrub typhus. (**B**) Histogram for significance assessment of Moran’s *I*.

**Figure 8:**
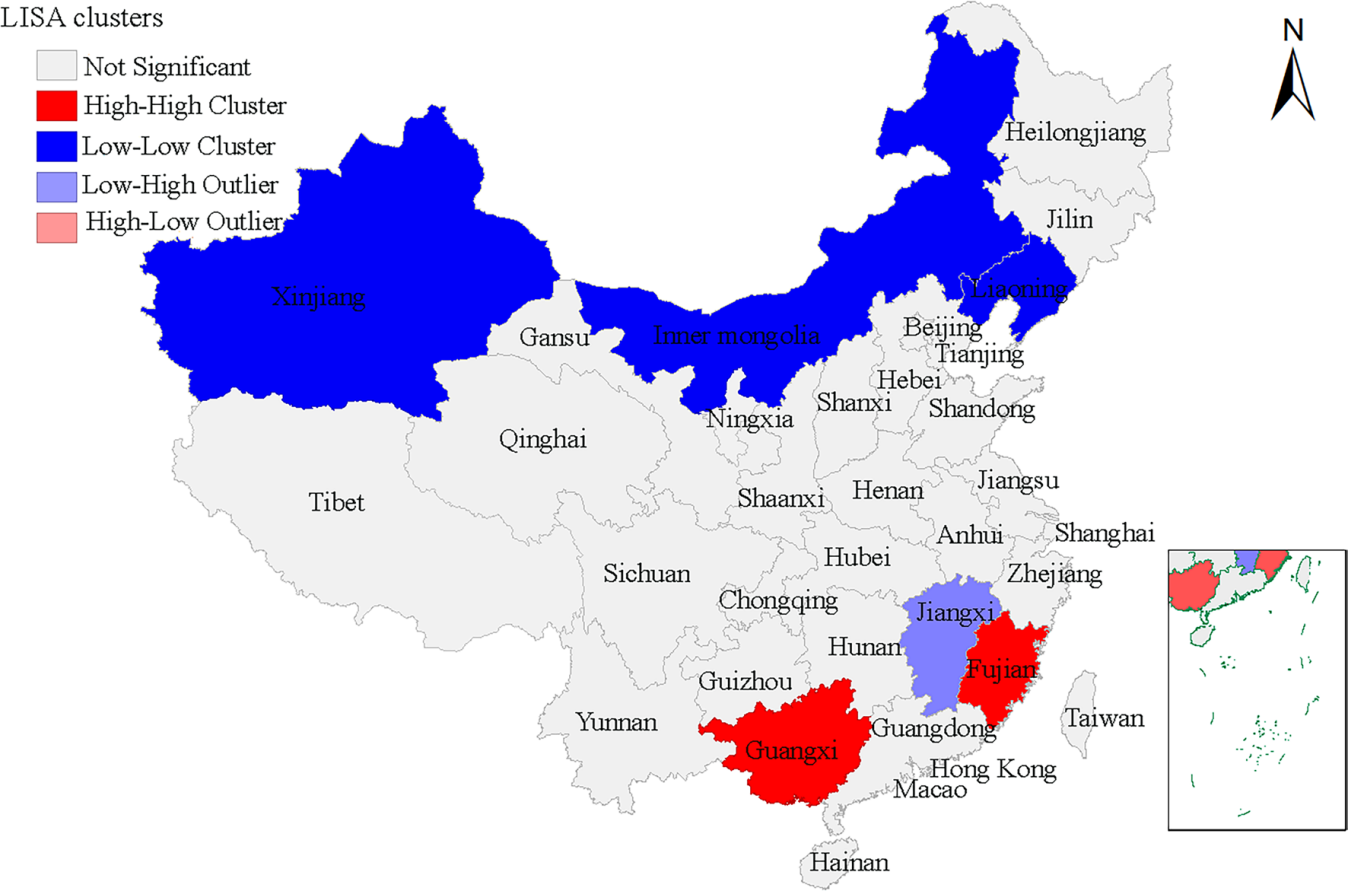
Local indicators of spatial association (LISA) cluster maps for the average annual scrub typhus incidence from 2006 to 2018 in China. This map was created by ArcGIS software (version 10.6, ESRI Inc.; Redlands, CA, USA). Homepage of ArcGIS software was https://www.esri.com/.

Moran’s scatter plot and the significance assessment by permutation test of spatial autocorrelation for annualized average incidence of scrub typhus in China is presented in Fig. 7. The value of global Moran’s *I* statistic (0.533) is shown in Fig. 7A, and the number of permutations (999) and *Z* scores (6.8056) are shown in Fig. 7B. The spatial autocorrelation analysis showed that a spatial positive correlation existed in the prevalence of scrub typhus on a national scale, which had the characteristic of aggregated distribution (*I* = 0.533, *P* < 0.05) (Fig. 7). The hotspots (High–High cluster area) and outliers of scrub typhus transmission in mainland China were identified through LISA analysis. LISA spatial cluster map shows the center of the cluster in color. High–High cluster indicates a statistically significant cluster of high scrub typhus incidence values. LISA analysis showed hotspots (High–High) were primarily located in the southern and southwestern provinces of China, such as, Yunnan, Guangxi, Guangdong and Fujian etc., and the geographical area expanded annually. Hotspots also occurred and expanded in the northern provinces of Anhui and Jiangsu (Fig. 8).

## Discussion

Since 1957, when scrub typhus was included in the Chinese notifiable disease monitoring system, the number of annually reported cases was approximately 1000 before 1986 in China, and then rose to > 2000 between 1986 and 1989^16^. Before 1986, scrub typhus was limited to south of the Yangtze River, from Zhejiang and Hunan in the north to Yunnan and Sichuan in the west, Hainan in the south, and Taiwan in the east. However, the regional distribution has expanded northward since then. Until 2014, all of the provinces in China except Qinghai reported scrub typhus cases^11^. There was a combined total of 182,991 scrub typhus cases during the time periods of 1952–1989 and 2006–2018, which were distributed in all of the provinces (31 provinces or municipalities) in mainland China. The amplitude and the magnitude of scrub typhus outbreaks in China remained rapid increase during the entire study period. Two main reasons may have contributed to this result. Firstly, as a natural-focus disease, scrub typhus had been paid too little attention where it was previously unknown^10^. Following the discovery of the first confirmed cases of scrub typhus in China, enhanced awareness of scrub typhus directly resulted in increased surveillance, coupled with the improvement of the diagnostic tests, and resulted in more cases being diagnosed and reported^17^. Secondly, it’s likely that rodents bearing infected mites may expand their ranges as climate changes, urbanization and globalization^18^.

In addition, the expansion of scrub typhus foci may be associated with secondary factors of human activities and environment change. For example, Deforestation, defined as land cover change from forest to non-forest regions, could be a human activities risk factor. The deforestation-induced secondary growth of scrub vegetation would provide a suitable environment for rodents, which are the natural hosts of vector mites, and may increase the density of mites^19,20^. Moreover, socio-economic factors, as a secondary cause, could have also served as important drivers for the transmission of scrub typhus in recent years. For example, the urbanization and change of land use may contribute to the spread of scrub typhus into urban areas by providing suitable habitats such as clearings, grasslands, and riverbanks for vectors and small rodents.

Scrub typhus is widespread in rural areas, which is similar to the patterns of geographic distribution in Southeast Asia^21,22^. The high endemicity in rural areas has been associated with people in rural areas who are exposed to environmental factors such as crops, bushes, animal pets and rodents^23^. Farmers constituted the majority of cases. Agricultural activities would increase the exposure to pathogen-carrying chigger mites^19,23,24^. For example, during the months of August to October, farmers are involved in harvesting activity in the fields, where they are exposed to the bites of larval mites. Notably, the disease was found to be prominent in elderly farmers, which may be associated with the rapidly changing demographics of rural areas in China. In modern China, young adults from rural areas often work as laborers in urban areas, leaving the elderly in their hometown taking care of grandchildren as well as farming^15,25^.

**Figure 9:**
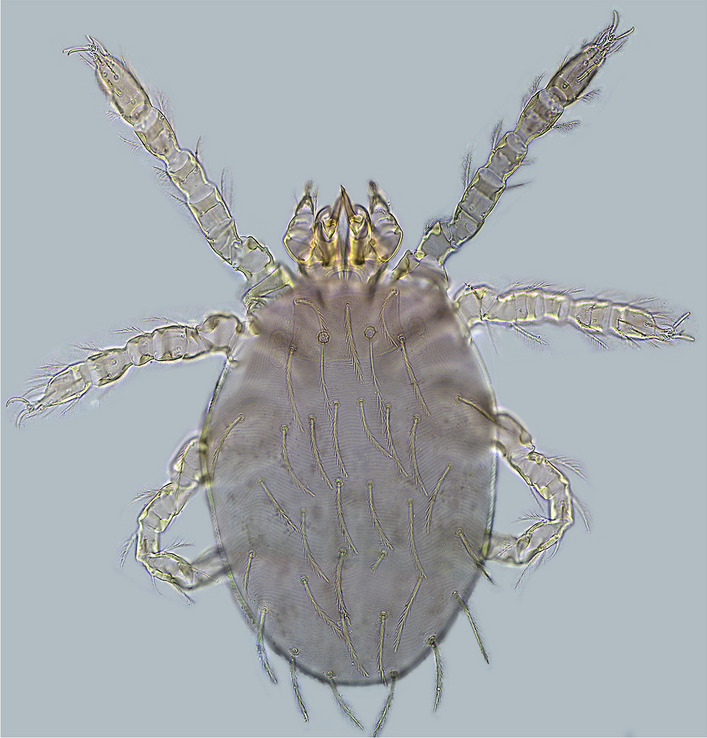
The photos of *L. deliense* (under microscope 10 × 40).

**Figure 10:**
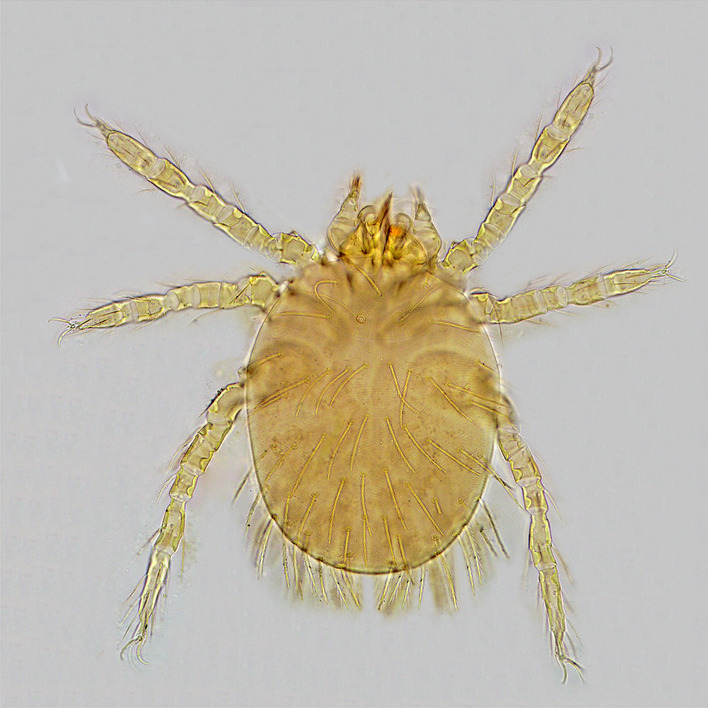
The photos of *L. scutellare* (under microscope 10 × 40).

The seasonal characteristics of scrub typhus in this study were similar to results in previous studies abroad. For example, in Japan the overall seasonality by month of onset was bimodal; a larger peak occurred from October to December and a smaller peak occurred from April to June^26,27^. In Korea, the incidence rate starts to increase from September, hitting a peak in October and November and sharply decreasing in December^28,29^. However, in Bhutan, there were virtually no cases between December and June, followed by a sharp increase in July with a peak of incidence in September, and a sudden drop from October onwards^30^. The difference of peak period can be explained partially by the types of chigger mites and life-cycle of the vector mites^26^. The occurrence of scrub typhus in Japan in spring/summer is reportedly due to *Leptotrombidium pallidum* and in autumn/winter is mainly due to *L. pallidum* and *L. scutellare*^31^. *L. scutellare* is the major cause of scrub typhus in Korea^32^*.* A study conducted by Zhang et al. concluded that there were four different seasonal patterns for scrub typhus in China: summer, winter, autumn–winter, and annually^16^. The seasonality south of the Yangtze River had a summer pattern while it had an autumn–winter pattern north of the Yangtze river. *L. deliense* (Fig. 9) was regarded as the main vector of summer type scrub typhus and *L. scutellare* (Fig. 10) was regarded as the main vector of autumn–winter type scrub typhus in mainland China^33,34^.

There was spatial clustering with hot spots in the southern provinces (Yunnan, Guangxi, Guangdong, Hainan, etc.) in our study results. Chiggers might indicate the connection between climate change and cases of scrub typhus. Chiggers are mainly located in grassy fields, gardens, parks, forests, bush, and moist areas around lakes or rivers, and their distribution is influenced by humidity, sunshine and temperature^35^. In the southern provinces of China, the climate is hot, humid and rainy, which is perfect for chigger mites. Moreover, the complicated topographic landform and high biodiversity of southern provinces may contribute to the extremely high species diversity of chigger mites in these provinces (especially Yunnan), which indirectly leads to a large number of scrub typhus cases^36^. The hot spots are largely concentrated in southern provinces, which may also be associated with societal changes. In recent years, the prosperity of ecotourism resulted in the development of southern regions in provinces such as Guangxi, Yunnan, and Guangdong, as well as a large increase of floating population in these provinces. This change in the population distribution could have increased the rate of human exposure to forests, riverbanks, and grassy regions that provide optimal environmental conditions for infected mites to thrive^37^. These hot spots should be targeted by policy-makers and local service providers for the establishment of refined disease control guidelines, including local vector control, health education, and promotion campaigns.

In our results, Guangdong and Yunnan provinces have the highest number of scrub typhus cases (38,439 cases in Guangdong and 35,420 cases in Yunnan). In recent years, a rising trend was observed, which caused a huge disease burden in Guangdong province. Several reasons might contribute to the increase of scrub typhus cases, such as variation of genotypes of *O. tsutsugamushi*^38^, increased risk of exposure to vegetation with more and more parks in the city construction^39^, improvement of the surveillance system, and climate change. A previous study showed that climate factors played an important role in the spread of vector-borne diseases^40^. Yunnan, an inland province at a low latitude and high elevation in southwestern China, has a vast territory with diversified and unique nature resources. The altitude of Yunnan ranges from the lowest in Hekou town (76.4 m) to the highest at the summit of Meili Snow Mountain (6740 m)^41,42^. Yunnan is also a very wide mountainous region with complicated weather conditions. The mean annual temperature in some river valleys and flatlands with low altitude (less than 500 m) is very high, which can exceed 30℃. The mean annual temperature in high mountainous regions with high altitude (more than 3500 m), however, is very low and even no summer season all the year^43,44^. There are abundant species of plants and animals with a plenty of biologically diverse gene resources in Yunnan, which has been one of the hot places of biodiversity conservation in China^45,46^. In addition, in Yunnan there are three rivers (Jinshajiang River, Lancangjiang River, and Nujiang River) flowing from the northwest towards the southwest. The three rivers parallel to each other, forming the “Protected Area of Three Parallel Rivers”, which is a famous “World Natural Heritage Area”. It is considered as a hotspot of biodiversity with high species diversity in Asian continent^47,48^. The geographical location with unique landscape, complex topography, and diversified ecological environment of Yunnan provide a good place to the survival of rodents and chigger mites. The rodent is an import intermediate host for the transmission of *Oriental tsutsugamushi*. The density and distribution of mites can directly influence the incidence of scrub typhus.

The three-dimensional trend surface analysis was done to frame the overall tendencies and to identify the outliers of the scrub typhus incidence in different geographical locations. In the present study, the LISA cluster maps revealed a higher incidence of scrub typhus infection in the southern provinces from 2006 to 2018, which agreed with the findings of the trend surface analysis.

Some limitations must be acknowledged for this study: First, the bias could exist in this study because all the surveillance data used were extracted from a passive reporting system, which indicated that some cases of scrub typhus might go underreported because of the nonspecific clinical symptoms. However, the data used in present research were the most comprehensive and reliable data on scrub typhus available at national level in China. Second, the present surveillance system was the lack of data on the accurate distribution of chigger mite species, causing it impossible to explore the association among human cases, hosts, pathogens and vectors.

## Methods

### Ethics statement

This study was approved by the institutional review boards (IRB) of Qujing Medical College (approval number: QJMCLL2023-110). Written informed consent was obtained from all patients in the study. All the data analyzed in this study were de-identified to protect patient confidentiality. All methods were carried out in accordance with relevant guidelines and regulations.

### Data collection and management

In China, scrub typhus is a vector-borne notifiable disease and physicians are required by law to report cases to the China Center for Disease Control and Prevention through the China Information System for Disease Control and Prevention (CISDCP). Scrub typhus case reports include basic demographic and clinical data including gender, age, occupation, residential address, date of onset of symptoms, laboratory diagnosis, and clinical outcome for each case. In this study, all data for scrub typhus cases in 1952–1989 and 2006–2018 were extracted from CISDCP. Demographic information, such as gender, age, occupation, and address, was not included, and there was no information regarding the geographical distribution, in the surveillance data from 1952 to 1989. All of the surveillance data between 2006 and 2018 included basic demographic and clinical information reported by physicians.

The case definition for scrub typhus consists of an individual who has traveled to an endemic area or reported contact with chigger mites or rodents within 3 weeks before the onset of illness, along with clinical manifestations (such as high fever, skin rash, lymphadenopathy and eschars or ulcers), and at least one of the following laboratory criteria: an agglutination titer ≥ 1:160 in the Weil-Felix test using the OX_K_ strain of *Proteus mirabilis*; a fourfold or more rise of antibody titer against *O. tsutsugamushi* using the indirect immunofluorescence antibody assay; detection of *O. tsutsugamushi* by PCR (polymerase chain reaction) in clinical specimens; or isolation of *O. tsutsugamushi* from clinical specimens^49^. Patients with other established causes of fever were excluded.

### Data analysis

The data were divided into two periods: phase I (1952–1989) and phase II (2006–2018). As no related data were obtained, the demographic characteristic analysis was not conducted during phase I, and the spatiotemporal distribution analysis was not included in phase I. All probable and confirmed cases were included in the analysis.

### Demographic distribution analysis

The demographic distribution characteristics including age, gender, occupation and geographic distribution of scrub typhus cases from 2006 to 2018 in China were analyzed according to surveillance data using χ^2^ test and Kruskal–Wallis *H* signed-rank test with SPSS v. 22.0 (SPSS Inc., USA), *P* < 0.05 was considered statistically significant.

### Trend surface analysis

Trend surface analysis is a multivariate statistical analysis method. It takes the incidence rates Z as the dependent variable and the two-dimensional plane coordinates X and Y as the independent variables. A multi-form (high-order) regression equation can be constructed to form a plane or more complex spatial surface in geometry to reflect the incidence rates in space^50,51^. Based on the theory of multiple regression analysis, a trend surface regression mathematical model was constructed. The latitude and longitude coordinates of different provinces (municipalities) and data of scrub typhus were collected in the model and the results were analyzed using ArcGIS software. This allowed a trend surface analysis chart to be drawn.

### Spatial autocorrelation analysis

A global spatial autocorrelation analysis and a Local Indicators of Spatial Association (LISA) analysis were adopted to analyze the spatial patterns and the potential hotspots associated with scrub typhus incidence. Global Moran’s *I* for global indication of spatial autocorrelation (GISA) reflects the similarity of attributes in spatially adjacent regions^52^. GISA was adopted to explore the global clustering characteristic of scrub typhus. Global Moran’s *I* is calculated as follows:

$$I=\frac{n}{\sum_{i=1}^{n}\sum_{j=1}^{n}{W}_{ij}}\times \frac{\sum_{i=1}^{n}\sum_{j=1}^{n}{W}_{ij}\left({x}_{i}-\overline{x }\right)\left({x}_{j}-\overline{x }\right)}{\sum_{i=1}^{n}{\left({x}_{i}-\overline{x }\right)}^{2}}$$

where n was the number of provinces (municipalities), *x*_*i*_ and *x*_*j*_ were the indicators of autocorrelations from unit index *i* and* j*, $\overline{x }$ was average and *W*_*i j*_ was the matrix of spatial weights. If unit *i* was adjoined to unit *j*, *W*_*i j*_ = 1; if not, *W*_*i j*_ = 0. Global Moran’s *I* Index, which ranged from − 1 to 1, reflects the similarity of attributes in spatially adjacent regions. Moran’s *I* Index = 0 implied a random spatial distribution. Moran’s *I* Index < 0 suggested a dispersing spatial distribution, and Moran’s *I* Index > 0 implied a clustering spatial distribution^53^.

Moran’s* I* is calculated on the basis of *Z*-test. *Z*-test statistics can be expressed as:

$$Z=\frac{|I-E\left(I\right)|}{\sqrt{Var\left(I\right)}}$$

In the above formula,* E* (*I*) represents the mathematically expected value of Moran's* I*; *Var* (*I*) represents the variance of Moran's *I*. Z score (> 1.96 or < − 1.96) means *P* < 0.05, which indicated that scrub typhus incidence was not distributed randomly and cases were likely to cluster.

Local Moran’s *I* for LISA is a measure of the similarity of difference between the attribute of the observation unit and those of surrounding units. It is calculated as follows:

$${I}_{i}=\frac{\left({x}_{i}-\overline{x }\right)}{{S}^{2}}\sum_{j=1}^{n}{W}_{ij}\left({x}_{j}-\overline{x }\right)$$

$${S}^{2}=\frac{\sum_{j=1, j\ne i}^{n}{W}_{ij}{\left({x}_{j}-\overline{x }\right)}^{2}}{n-1}$$

In the above formulae, *n*, *x*_*i*_, *x*_*j*_, *W*_*i j*_ and $\overline{x }$ are the same as in the former formula.

LISA was calculated to explore significant hot spots (High–High), cold spots (Low–Low), and outliers (High–Low and Low–High)^54^.

GISA analyses and LISA analyses can be realized by GeoDa0.9.5-*i*, a freely available spatial statistics software package (https://geodacenter.asu.edu/). Monte Carlo randomization was employed to assess the significance of Moran’s *I* and the number of permutation tests was set to 999. *Z* score (≥ 1.96) indicated that scrub typhus incidence was not distributed randomly and cases were likely to cluster.

## Data Availability

The datasets used and/or analyzed during the current study are available from the corresponding author on reasonable request Declarations.
